# The Educational Gradient in Intake of Energy and Macronutrients in the General Adult and Elderly Population: The Tromsø Study 2015–2016

**DOI:** 10.3390/nu13020405

**Published:** 2021-01-28

**Authors:** Linn Nilsen, Laila A. Hopstock, Guri Skeie, Sameline Grimsgaard, Marie W. Lundblad

**Affiliations:** Department of Community Medicine, UiT The Arctic University of Norway, 9037 Tromsø, Norway; linn.nilsen@uit.no (L.N.); laila.hopstock@uit.no (L.A.H.); guri.skeie@uit.no (G.S.); sameline.grimsgaard@uit.no (S.G.)

**Keywords:** public health, socioeconomic status, educational gradient, diet, energy intake, macronutrient intake, population-based studies, Norway

## Abstract

Worldwide, there are socioeconomic inequalities in health and diet. We studied the relationship between education and nutrient intake in 11,302 women and men aged 40–96 years who participated in the seventh survey of the population-based Tromsø Study (2015–2016), Norway (attendance 65%). Diet was assessed using a validated food-frequency questionnaire. We examined the association between education and intake of total energy and macronutrients by sex using linear and logistic regression models adjusted for age, body mass index, leisure time physical activity and smoking. The intake of macronutrients was compared with the Nordic Nutrition Recommendations 2012. There was a positive association between education and intake of fiber and alcohol, and a negative association between education and intake of total carbohydrates and added sugar in both women and men. Participants with long tertiary education had higher odds of being compliant with the recommended intake of fiber and protein and the maximum recommended level for added sugar and had lower odds of being compliant with the recommended intake of total carbohydrates and the maximum recommended level for alcohol, compared to participants with primary education. Overall, we found that participants with higher education were more compliant with the Nordic Nutrition Recommendations 2012.

## 1. Introduction

Socioeconomic factors cause inequalities in health worldwide [1]. Those with lower socioeconomic status (SES) have higher overall mortality, morbidity and prevalence of risk factors compared to those with higher SES [1,2,3,4,5,6]. Reduction in social inequality in health is central to the United Nation’s Sustainable Development Goals [7] and World Health Organization’s Global Action Plan for the Prevention and Control of Non-Communicable Diseases 2013–2020 [8]. In 2017, 27% of all worldwide deaths related to non-communicable diseases were attributable to dietary risk factors [9]. A social gradient is also observed in diet. Hence, it is important to reduce social inequality in diet, and population surveillance surveys are crucial to evaluate progress and propose actions towards the goal. Dietary surveys from Nordic countries have previously shown a positive association between education and the intake of vegetables, fruits and berries, grain products, fiber and alcohol, and a negative association with the intake of red meat and total fat [10,11,12,13].

The Nordic Nutrition Recommendations 2012 (NNR 2012) is a collaboration between the Nordic countries and forms the scientific foundation for recommendations, guidelines and nutritional policies in Norway, Sweden, Finland, Denmark and Iceland [14]. Dietary surveys presenting diet in groups of SES from Nordic countries conducted in 2010–2013 showed inconsistent results, and there is a need for more recent data [10,15,16,17]. The aim of this study is to examine the educational gradient in the intake of total energy and macronutrients and the compliance with NNR 2012 in a large population-based study of Norwegian adults and elderly.

## 2. Materials and Methods

### 2.1. Study Population and Data Collection

The Tromsø Study is an ongoing population-based cohort study in the municipality of Tromsø, Norway. In the seventh survey (Tromsø 7) conducted in 2015–2016, all inhabitants aged 40 years and older (*N* = 32,591) were invited. A total of 21,083 participants aged 40–99 years attended (65%) [18].

Data collection included questionnaires, biological sampling and clinical examinations. An extensive and previously validated [19,20] food frequency questionnaire (FFQ) was used to measure food and nutrient intake (details previously described in Lundblad et al. [21]). A full version of the FFQ is available on the Tromsø Study webpage [18]. Calculation of food, macro- and micronutrient intake was performed at the University of Oslo using the food and nutrient calculation system Kostberegningssystemet (KBS), database AE14 (based on the Norwegian food composition tables 2014 and 2015 [22]), in software version 7.3. We included total energy in megajoule (MJ) per day and the following macronutrients in energy percentage (E%) or grams per day (g/day): total carbohydrates (E%), added sugar (E%; defined as refined or industrially manufactured sugar added during industrial production or preparation in the home [22]), fiber (g/day), protein (E%), total fat (E%), saturated fat (SFAs) (E%), monounsaturated fat (MUFAs) (E%), polyunsaturated fat (PUFAs) (E%) and alcohol (E%). Data on educational level (primary/up to ten years), secondary (a minimum of three additional years), short tertiary (college/university less than four years) and long tertiary (college/university four years or more); leisure-time physical activity level (sedentary, light, moderate and vigorous), and smoking status (never, previous, current) were included from questionnaires. Physical activity was reported on a four-level scale based on the Saltin and Grimby questionnaire [23]. Body height and weight were measured by trained personnel at examination, and body mass index (BMI) was calculated as weight in kilograms (kg) divided by height in meters (m) squared (kg/m^2^). BMI was categorized into three groups: normal weight (BMI < 25.0 kg/m^2^), overweight (BMI 25.0–29.9 kg/m^2^) and obesity (BMI ≥ 30.0 kg/m^2^). Participants who were underweight (BMI < 18.5 kg/m^2^) were few (*n* = 48) and, therefore, merged with the normal-weight category.

A total of 15,146 participants aged 40–96 years returned the FFQ (response 72% for participants who attended Tromsø 7). In accordance with Lundblad et al. [21], participants that completed less than 90% of the FFQ (*n* = 3489) and participants with highly unrealistic energy intakes (the 1% with the lowest/highest absolute energy intake; below 3.95 MJ or above 21.3 MJ per day, respectively) (*n* = 232) were excluded. Additionally, all participants with missing data on education (*n* = 123) were excluded. Finally, a total of 11,302 participants were included in the analysis. This equals 54% of all participants in Tromsø 7 and 75% of all participants who returned the FFQ.

### 2.2. Statistical Analysis

Differences in participant characteristics between education levels were tested by one-way ANOVA (for continuous variables) and Pearson’s chi-square test (for categorical variables) (Table 1).

We used descriptive analyses to present the median (25th–75th percentile) intake of energy and the energy providing macronutrients’ carbohydrates (including added sugar and fiber), proteins, fat (including subgroups SFAs, MUFAs and PUFAs) and alcohol, and to compare the intakes with NNR 2012 (Table 2). Descriptive analyses were also used to present the proportion of participants that were compliant with the respective nutrient recommendation, as well as the proportion above/below the recommended intake for nutrients where the recommendation is a range, overall and in different education levels (Table 2).

Multiple linear and binary logistic regression was used to calculate the adjusted effect of educational level on intake of energy and each of the macronutrients (Table 3) and the odds ratio (OR) of being compliant with the NNR2012 at different levels of education (Table 4). Education and potential confounders (age groups, BMI groups, physical activity level and smoking status) were included as independent variables with the lowest level as reference. The linear trends in education were assessed by including education as a continuous variable in an identical analysis (Table 3 and Table 4).

We used Student´s t-test (for continuous variables) and Pearson´s chi-square test (for categorical variables) to examine potential differences according to mean age and BMI and the distribution of participants in groups of sex, age, BMI, educational level, physical activity level and smoking status in the included versus the excluded participants. (Table S1).

Due to well-known sex differences in the intake of energy and nutrients [14], all analyses were stratified by sex. All analyses were performed using IBM SPSS 26 for Mac (IBM Corp. Released 2019. IBM SPSS for Macintosh, Version 26.0.0.1. Armonk, NY: IBM Corp). The significance level was set to 5% for all tests.

### 2.3. Ethical Considerations and Data Safety

Tromsø 7 data collection was approved by the Regional Committee for Medical Research Ethics (REC North ref. 2014/940) and the Norwegian Data Protection Authority and performed in accordance with the 1964 Helsinki declaration and its later amendments. All participants gave written informed consent.

## 3. Results

### 3.1. Study Sample

About 60% of women and 75% of men had overweight or obesity (Table 1). More than 50% had received short or long tertiary education, and more than 50% reported light leisure-time physical activity. In total, 12.5% were smokers, and 42% were never smokers (Table 1). Participants with higher education had lower age and BMI, higher physical activity levels and a higher proportion were never smokers, compared to those with primary education (all; *p* < 0.001) (Table 1).

Participants included in the final sample had lower BMI (*p* < 0.001), higher education level (*p* < 0.001), a higher proportion were women (*p* < 0.05), and a lower proportion were current smokers (*p* < 0.001) and had sedentary leisure-time physical activity level, compared to participants who were excluded from the study (Table S1).

### 3.2. Women

In women, the median total energy intake was 8.5 MJ/day (Table 2). The median daily intake was 42 E% for total carbohydrates, 27 g for fiber, 5 E% for added sugar, 18 E% for proteins, 35 E% for total fat, 13 E% for SFAs and MUFAs, 6 E% for PUFAs and 2 E% for alcohol (Table 2). About 85% met the recommendation for proteins, total fat and MUFAs, 73% met the recommendation for PUFAs, 60% met the recommendations for fiber and 31% met the recommendation for total carbohydrates (Table 2). For the recommendations given as a range, the majority of those not meeting recommendations were above the recommended range for proteins and total fat, and below the recommended range for carbohydrates, MUFAs and PUFAs (Table 2). A total of 93, 81 and 15% reported intakes below the maximum recommended level for added sugar, alcohol and SFAs, respectively (Table 2). The intake of energy, fiber, total fat, MUFAs and alcohol were positively associated with education (*p* < 0.05) (Table 3). The intake of total carbohydrates and added sugar were negatively associated with education (*p* < 0.05) (Table 3). Women with higher education had higher odds of being compliant with recommendations for fiber (*p* < 0.05), and lower odds of being compliant with the recommendation for total carbohydrates, total fat and alcohol compared to women with primary education (Table 4).

### 3.3. Men

In men, the median total energy intake was 10.4 MJ/day (Table 2). For macronutrients, the median daily intake was 43 E% for total carbohydrates, 27 g for fiber, 5 E% for added sugar, 17 E% for proteins, 35 E% for total fat, 12 E% for SFAs and MUFAs, 6 E% for PUFAs and 3 E% for alcohol (Table 2). About 85% met the recommendations for proteins, total fat and MUFAs, 73% met the recommendation for PUFAs, 33% met the recommendation for total carbohydrates and 23% met the recommendation for fiber (Table 2). For the recommendations given as a range, the majority of those not meeting recommendations were above the recommended range for proteins and total fat, and below the recommended range for carbohydrates, MUFAs and PUFAs (Table 2). A total of 90, 72 and 16% reported intakes below the maximum recommended level for added sugar, alcohol and SFAs, respectively (Table 2). The intake of fiber and alcohol was positively associated with education level (*p* < 0.001) (Table 3). The intake of total carbohydrates, added sugar and SFAs was negatively associated with education level (*p* < 0.001, <0.001 and 0.009, respectively) (Table 3). Compared to men with primary education, men with higher education had higher odds of being compliant with recommendations for added sugar and lower odds of being compliant with recommendations for alcohol (*p* < 0.001) (Table 4).

## 4. Discussion

In this study, we found educational gradients in the reported intake of several macronutrients. Study participants with long tertiary education had higher odds of being compliant with the NNR2012 compared to women and men with primary education. However, the observed differences were small, and the clinical relevance is unclear.

Median intakes of energy and macronutrients found in this study were similar to what was observed in the Norwegian national dietary survey NORKOST 3 from 2010–2011, although the data collection method and sample age range differed [10]. We found a positive association between education level and reported intake of fiber and alcohol in both women and men, and for energy intake, total fat and MUFAs in women. A negative association was found between education level and reported intake of total carbohydrates and added sugar in women and men, for SFAs in men and for protein intake in men with long tertiary education. Similar results have been found in other Nordic studies [10,15,16,24]. An individual’s requirement of energy and nutrients depends on sex, age, body size and physical activity level [14]. These are all treated as confounders and adjusted for in the present study; thus, the educational gradients observed were not explained by the included determinants of energy and nutrient requirements.

This study had considerable statistical power due to the large number of participants and several analyses showed statistically significant results that may not be clinically relevant. The higher energy intake found in women with long tertiary education compared to those with primary education represents one apple or two squares of milk chocolate (20 g) per day. This may seem like a minor difference, but accumulated over a year, such an excess energy intake may result in a weight gain of approximately 3 kg per year [14]. Additionally, one extra apple gives extra fiber, beta-carotene and vitamin C, while two squares of milk chocolate give extra SFAs and added sugar. Thus, the type of foods the energy comes from is of great importance. The difference in fiber intake between the highest- and the lowest-educated men represents three tablespoons of oatmeal per day. These are differences that might seem small and irrelevant on daily, monthly or even yearly basis. However, throughout a lifespan, this might lead to social differences in health profiles. Other results are obviously both significant and clinically relevant. The added sugar intake in those with long tertiary education is one E% lower than in those with primary education. The maximum recommended level for added sugar is 10 E%, and the intake in all educational groups is below this [14].

The dietary gradients found in this study demonstrates a need to improve the health literacy of the population. Health and dietary information, e.g., nutrient recommendations and food-based dietary guidelines, need to be wider distributed and communicated in a way that is understandable, applicable and feasible for everyone. Health communication should be emphasized in the education of healthcare personnel such as nurses, medical doctors, nutritionists and public health workers. We need to take extra measures to reach all members of the community with tailored information, with emphasis on vulnerable groups (e.g., those with low education). Pricing mechanisms, through lower prices on healthy foods such as vegetables, fruits, berries and fish and higher prices on foods rich on added sugar and SFAs would undoubtedly also be efficient [25].

### Strengths and Limitations

This study had a population-based design, a large number of participants and a high participation rate. It included participants from both urban and rural living areas. The population is similar to the general Norwegian population in regard to the distribution of sex, age, educational attainment and the proportion of current smokers [26,27,28]. However, as in all population-based studies, there is a risk of selection bias. Previous analyses from Norwegian health surveys have shown that participants more often tend to be married, have better health and higher education than the non-responders [29,30,31]. Results from the analysis of responders versus the non-responders of the FFQ in Tromsø 7 showed an overrepresentation of women, participants aged 60–69 years, participants with normal BMI, tertiary education (short and long) and moderate physical activity among responders compared to non-responders (Table S1). It is possible that the inclusion criteria of only including participants who answered 90% or more of the FFQ has contributed to a selection bias. Among the included participants, there was a higher proportion of participants with tertiary education and a lower proportion of participants with primary and secondary education. Thus, there might be a positive educational gradient in the overall completeness of the FFQ. We did, however, examine whether completeness of FFQ differed in different educational levels among those included in the final sample (results not shown). Independent of educational level, all participants answered approximately 94% of the FFQ, and we, therefore, assume that there was no educational gradient in the overall completeness of the FFQ among the included participants.

The design and analyses used in this study allowed us to investigate the educational gradient in diet with adjustments for potential confounders and by investigating two important aspects of the diet: the absolute intake of nutrients and the compliance with NNR 2012 in levels of education. It is plausible that over- and underreporting have influenced the results. Studies have shown that underreporting of unhealthy foods is more frequent among women, those with higher BMI, smokers and those with lower education, and that fatty foods such as cake, cheese, spreads, milk and snacks are among the food items that are most likely to be underreported [32].

Educational level is a common indicator frequently used for SES. However, it is possible that other indicators, or a combination of indicators, can give a more precise definition of the exposure. A Norwegian report on education and inequalities in health concluded that education as an SES indicator is a practical and appropriate indicator to rank individuals in the socioeconomic hierarchy [33]. They also concluded that education is not always the best indicator for all types of health outcomes, as different indicators show different associations with outcomes [33]. A study of 91,900 participants in France examined the independent effect of the SES indicators education, income and occupation on nutrient intake [34]. The main findings were that the different indicators were associated with specific differences in nutrient intake, suggesting that they underpin different social processes. The authors concluded that education is an important driver of nutrient intake in the lower SES groups [34]. Thus, using only education level as an SES indicator in the present study is a potential weakness.

The dietary assessment method used in the present study has several strengths. Firstly, the FFQ used was comprehensive and validated for several dietary components [19,20,35]. Secondly, the FFQ mapped the average diet during the previous year, and not only during the previous day or week. Thirdly, we did, in accordance with previous studies [13,21,36,37,38], exclude participants with incomplete FFQs. This ensured that intentionally unfilled questionnaires were excluded. However, when using an FFQ, there is always a risk of information bias, especially differential misclassification. As the FFQ is self-reported, it is unlikely that the participants remember their diet over the past year with 100% accuracy. We cannot exclude that some food items are systematically over- or underreported. Social desirability bias, i.e., “the tendency of some respondents to report an answer in a way they deem to be more socially acceptable than would be their ‘true’ answer”, may result in underreporting of fatty and sugary foods and overreporting of healthy foods such as vegetables, fruits and berries [39].

## 5. Conclusions

This study shows educational gradients in the intake of energy and macronutrients, mostly in favor of participants with higher education, with the exception of alcohol. A positive educational gradient was found for the intake of fiber and alcohol, and a negative educational gradient was found for total carbohydrates and added sugar in both women and men. Further, a positive gradient was found for energy, total fat and MUFAs in women, and a negative gradient was found for SFAs in men. This study provides valuable knowledge regarding SES and diet and may serve as an important benchmark for efforts towards reducing socioeconomic inequalities in health and diet.

## Figures and Tables

**Table 1:** Characteristics of study sample by sex and education level. The Tromsø Study 2015–2016.

		Total	Education ^a^			
			1	2	3	4
**Women**						
*n* (%)		6043	1270 (21.0)	1539 (25.5)	1112 (18.4)	2122 (35.1)
Age (mean (SD)) (years)		56.8 (10.7)	64.4 (10.1)	57.1 (10.0)	55.0 (9.9)	53.0 (9.5)
Age group (%)	40–49 years	30.2	8.7	27.0	35.3	42.9
	50–59 years	29.8	20.8	32.7	32.3	31.6
	60–69 years	26.9	39.1	28.5	23.4	20.4
	70–79 years	10.9	25.2	10.1	8.0	4.5
	80–96 years	2.1	6.2	1.8	1.1	0.5
BMI (mean (SD)) (kg/m^2^)		26.8 (4.9)	27.7 (21.5)	27.4 (5.1)	26.5 (4.6)	26.0 (4.6)
BMI Group ^b^ (%)	Normal	39.9	30.3	34.1	42.3	48.7
	Overweight	37.8	41.8	40.0	37.7	33.8
	Obesity	22.0	27.4	25.7	20.0	17.2
Physical activity level ^c^ (%)	Sedentary	12.3	19.0	14.0	10.8	8.9
	Light	63.5	67.0	68.6	66.4	60.6
	Moderate	19.6	13.3	16.4	20.6	26.4
	Vigorous	2.3	0.7	1.1	2.3	4.1
Smoking status (%)	Current	13.5	19.1	18.6	11.5	7.4
	Previous	44.0	48.9	47.3	43.7	38.9
	Never	42.0	31.1	33.7	44.3	53.3
**Men**						
*n* (%)		5259	1057 (20.1)	1525 (29.0)	1209 (23.0)	1468 (27.9)
Age (mean (SD)) (years)		58.0 (10.9)	62.2 (10.4)	57.8 (11.0)	57.5 (10.6)	55.6 (10.5)
Age group (%)	40–49 years	27.2	13.7	28.0	26.7	36.3
	50–59 years	27.7	24.5	28.1	31.0	26.8
	60–69 years	28.6	36.5	27.0	27.8	25.1
	70–79 years	14.2	20.9	14.4	12.2	10.7
	80–96 years	2.4	4.4	2.5	2.3	1.0
BMI (mean (SD)) (kg/m^2^)		27.6 (3.9)	28.1 (4.2)	28.0 (4.0)	27.8 (3.8)	26.8 (3.6)
BMI Group ^b^ (%)	Normal	25.4	23.3	22.0	22.8	32.5
	Overweight	50.7	48.0	51.9	51.3	51.1
	Obesity	23.7	28.4	25.9	25.7	16.3
Physical activity level ^c^ (%)	Sedentary	13.2	19.2	15.0	10.9	9.9
	Light	50.9	53.9	53.8	52.4	47.5
	Moderate	30.6	25.4	28.6	33.1	35.9
	Vigorous	3.8	1.6	2.6	3.7	6.8
Smoking status (%)	Current	11.4	17.9	13.8	9.4	6.0
	Previous	45.3	54.0	49.8	44.7	34.9
	Never	42.8	27.6	35.9	45.3	58.8

Overall differences between education levels were tested by one-way ANOVA and Pearson’s chi-square test and were statistically significant for all characteristics (*p* < 0.001). SD, standard deviation. BMI, body mass index. ^a^ 1: Primary (up to 10 years of schooling), 2: upper secondary education (a minimum of 3 years), 3: tertiary education, short: college/university less than 4 years, 4: tertiary education, long: college/university 4 years or more. ^b^ Normal (BMI < 25.0 kg/m^2^), overweight (BMI 25.0–29.9 kg/m^2^), obesity (BMI ≥ 30 kg/m^2^). ^c^ Exercise and physical activity in leisure time over the last year. Sedentary: reading, watching TV/screen or other sedentary activity, light: walking, cycling or other forms of exercise at least 4 h a week, moderate: participation in recreational sports, heavy gardening, snow shoveling, etc., at least 4 h a week, Vigorous: participation in hard training or sports competitions, regularly several times a week.

**Table 2:** Intake of energy and nutrients by sex and education level, and compliance with Nordic Nutrition Recommendations 2012. The Tromsø Study 2015–2016.

	NNR 2012	Median Intake (25th–75th Percentile)	Compliant (%) (Below/Above)				
			All	Education ^a^			
				1	2	3	4
**Women**							
Energy (MJ/day)	-	8.5 (7.0, 10.4)	-				
Total carbohydrates (E%)	45–60 E%	42 (38–46)	31 (69/0.3)	37 (63/0.6)	30 (70/0.3)	29 (71/0.4)	30 (70/0.1)
Fiber (g/day)	≥25 g/day	27 (22–34)	60	52	58	61	65
Added sugar (E%)	<10 E%	4.8 (3.2–6.8)	93	91	91	92	94
Proteins (E%)	10–20 E%	17.7 (16.1–19.4)	83 (0.1/17)	80 (0.3/20)	81 (0.1/20)	84 (0.1/16)	85 (0/15)
Total fat (E%)	25–40 E%	35 (31–38)	81 (3/16)	85 (4/11)	81 (3/16)	79 (3/19)	79 (3/18)
Saturated fat (E%)	<10 E%	12.4 (10.8–14.2)	15	15	15	15	14
Monounsaturated fat (E%)	10–20 E%	12.8 (11.1–14.5)	86 (12/2)	83 (17/0.4)	87 (11/2)	87 (11/3)	89 (10/2)
Polyunsaturated fat (E%)	5–10 E%	5.8 (4.9–6.9)	73 (25/2)	72 (27/1)	75 (23/2)	73 (24/2)	73 (26/2)
Alcohol (E%)	<5 E%	1.8 (0.5–4.1)	81	87	82	79	77
**Men**							
Energy (MJ/day)	-	10.4 (8.4–12.5)	-				
Total carbohydrates (E%)	45–60 E%	43 (39–46)	33 (69/0.3)	35 (65/0.6)	34 (66/0.2)	30 (67/0.2)	33 (66/0.2)
Fiber (g/day)	≥35 g/day	27 (22–34)	23	52	59	63	67
Added sugar (E%)	<10 E%	4.9 (3.3–7.2)	90	86	89	92	92
Proteins (E%)	10–20 E%	17.3 (15.7–19.0)	86 (0.2/14)	83 (0.2/17)	85 (0.4/15)	86 (0/14)	91 (0.2/9)
Total fat (E%)	25–40 E%	35 (31–378)	83 (4/13)	82 (5/13)	83 (5/13)	83 (5/12)	83 (5/13)
Saturated fat (E%)	<10 E%	12.3 (10.7–13.9)	16	16	15	16	18
Monounsaturated fat (E%)	10–20 E%	12.4 (10.9–14.0)	85 (14/1)	82 (17/1)	87 (12/1)	86 (12/1)	85 (13/1)
Polyunsaturated fat (E%)	5–10 E%	5.9 (5.0–7.0)	73 (25/2)	69 (28/3)	75 (23/3)	78 (21/1)	71 (27/1)
Alcohol (E%)	<5 E%	2.7 (1.0–5.4)	72	78	75	69	66

E%, proportion of total energy intake. NNR 2012, Nordic Nutrition Recommendations 2012. ^a^ 1: Primary education (up to 10 years), 2: upper secondary education (minimum 3 years), 3: tertiary education, short: college/university less than 4 years, 4: tertiary education, long: college/university 4 years or more.

**Table 3:** Adjusted differences in energy and nutrient intakes by sex and education level. The Tromsø Study 2015–2016.

	Education ^a^				*p* Linear Trend
	1 (ref.)	2	3	4	
**Women**					
Energy (MJ/day)	8.6	0.36 ** (0.15, 0.57)	0.36 * (0.13, 0.59)	0.43 ** (0.22, 0.64)	<0.001
Total carbohydrates (E%)	45.4	−1.36 ** (−1.83, −0.89)	−1.71 ** (−2.24, −1.19)	−2.02 ** (−2.50, −1.54)	<0.001
Fiber (g/day)	23.0	1.57 ** (0.84, 2.31)	1.59 ** (0.77, 2.40)	2.19 ** (1.45, 2.94)	<0.001
Added sugar (E%)	7.3	−0.34 * (−0.61, −0.07)	−0.62 ** (−0.91, −0.33)	−0.86 ** (−1.13, −0.60)	<0.001
Proteins (E%)	16.5	0.009 (−0.18, 0.20)	−0.17 (−0.39, 0.04)	−0.13 (−0.33, 0.06)	0.08
Total fat (E%)	34.8	0.58 * (0.14, 1.02)	0.73 * (0.24, 1.21)	0.59 * (0.15, 1.03)	0.03
Saturated fat (E%)	13.2	−0.06 (−0.27, 0.15)	0.03 (−0.20, 0.26)	0.12 (−0.12, 0.31)	0.2
Monounsaturated fat (E%)	12.7	0.46 ** (0.25, 0.67)	0.53 ** (0.30, 0.66)	0.44 ** (0.23, 0.66)	<0.001
Polyunsaturated fat (E%)	5.9	0.11 (−0.008, 0.23)	0.08 (0.05, 0.21)	−0.04 (−0.16, 0.07)	0.1
Alcohol (E%)	1.0	0.75 **(0.50, 1.00)	1.13 ** (0.85, 1.41)	1.50 ** (1.25, 1.76)	<0.001
**Men**					
Energy (MJ/day)	10.9	0.03 (−0.21, 0.27)	0.02 (−0.23, 0.28)	0.12 (−0.13, 0.38)	0.4
Total carbohydrates (E%)	45.1	−0.59 * (−1.06, −0.12)	−1.37 ** (−1.87, −0.87)	−1.22 ** (−1.72, −0.73)	<0.001
Fiber (g/day)	25.3	0.63 (−0.14, 1.39)	1.12 * (0.31, 1.93)	1.81 ** (1.00, 2.61)	<0001
Added sugar (E%)	7.9	−0.47 * (−0.75, −0.19)	−0.83 ** (−1.13, −0.54)	−0.89 ** (−1.18, −0.60)	<0.001
Proteins (E%)	16.2	0.06 (−0.13, 0.25)	0.12 (−0.08, 0.32)	−0.27 * (−0.47, −0.07)	0.007
Total fat (E%)	35.5	0.04 (−0.41, 0.49)	0.03 (−0.45, 0.51)	−0.30 (−0.77, 0.17)	0.2
Saturated fat (E%)	13.6	−0.26 * (−0.47, −0.06)	0.34 * (−0.56, −0.12)	−0.31 * (−0.53, 0.09)	0.009
Monounsaturated fat (E%)	12.9	0.20 (−0.007, 0.41)	0.28 * (0.06, 0.50)	0.14 (−0.08, 0.36)	0.2
Polyunsaturated fat (E%)	6.1	0.07 (−0.06, 0.19)	0.03 (−0.10, 0.17)	0.17 * (−0.30, −0.03)	0.004
Alcohol (E%)	1.4	0.45 * (0.11, 0.78)	1.15 ** (0.80, 1.51)	1.68 ** (1.33, 2.03)	<0.001

Results from linear regression analysis given as unstandardized B (95% CI). Adjusted for age groups (40–49 years (reference)/50–59 years/60–69 years/70–79 years/80–96 years), body mass index groups (normal (reference)/overweight/obesity), physical activity level (sedentary (reference)/light/moderate/vigorous) and smoking status (never smoker (reference)/current smoker/previous smoker). * *p*-value for linear regression analysis <0.05. ** *p*-value for linear regression analysis <0.001. CI, confidence interval. E%, proportion of total energy intake. **^a^** 1: Primary education (up to 10 years), 2: upper secondary education (minimum 3 years), 3: tertiary education, short: college/university less than 4 years, 4: tertiary education, long: college/university 4 years or more.

**Table 4:** Adjusted odds of compliance with the Nordic Nutrition Recommendations 2012. The Tromsø Study 2015–2016.

	Education ^a^				*p* Linear Trend
	1 (ref.)	2	3	4	
**Women**					
Total carbohydrates (45–60 E%)	1.0	0.7 ** (0.6, 0.9)	0.6 ** (0.5, 0.8)	0.6 ** (0.5, 0.8)	<0.001
Fiber (≥25 g/day)	1.0	1.2 * (1.0, 1.4)	1.3 * (1.08, 1.5)	1.5 ** (1.3, 1.8)	<0.001
Added sugar (<10 E%)	1.0	1.1 (0.8, 1.5)	1.3 (0.9, 1.8)	1.8 ** (1.3, 2.4)	<0.001
Proteins (10–20 E%)	1.0	1.0 (0.8, 1.2)	1.2 (1.0, 1.5)	1.3 ** (1.1, 1.7)	<0.001
Total fat (25–40 E%)	1.0	0.8 * (0.6, 1.0)	0.6 ** (0.5, 0.8)	0.7 ** (0.5, 0.8)	<0.001
Saturated fat (<10 E%)	1.0	1.0 (0.8, 1.2)	0.9 (0.7, 1.2)	0.8 (0.7, 1.0)	0.09
Monounsaturated fat (10–20 E%)	1.0	1.2 (1.0, 1.5)	1.1 (0.7, 1.3)	1.2 (1.0, 1.5)	0.2
Polyunsaturated fat (5–10 E%)	1.0	1.1 (0.9, 1.3)	1.0 (0.8, 1.2)	0.9 (0.8, 1.1)	0.2
Alcohol (<5 E%)	1.0	0.6 ** (0.5, 0.7)	0.4 ** (0.3, 0.5)	0.3 ** (0.3, 0.4)	<0.001
**Men**					
Total carbohydrates (45–60 E%)	1.0	0.9 (0.8, 1.0)	0.8 ** (0.6, 0.8)	0.7 * (0.6, 0.9)	<0.001
Fiber (≥35 g/day)	1.0	1.0 (0.8, 1.3)	1.1 (0.9,1.4)	1.4 ** (1.2, 1.8)	<0.001
Added sugar (<10 E%)	1.0	1.3 * (1.0, 1.6)	2.0 ** (1.5, 2.6)	2.2 ** (1.6, 2.8)	<0.001
Proteins (10–20 E%)	1.0	1.0 (0.8, 1.3)	1.2 (0.9, 1.5)	1.6 ** (1.2, 2.0)	<0.001
Total fat (25–40 E%)	1.0	1.1 (0.9, 1.3)	1.1 (0.8, 1.4)	1.0 (0.8, 1.3)	0.8
Saturated fat (<10 E%)	1.0	1.0 (0.8, 1.2)	1.0 (0.8, 1.3)	1.2 * (1.0, 1.6)	0.02
Monounsaturated fat (10–20 E%)	1.0	1.3 * (1.1, 1.7)	1.3 * (1.0, 1.7)	1.1 (0.9, 1.4)	0.4
Polyunsaturated fat (5–10 E%)	1.0	1.3 * (1.1, 1.5)	1.5 ** (1.2, 1.8)	1.0 (0.9, 1.3)	0.7
Alcohol (<5 E%)	1.0	0.8 * (0.6, 0.9)	0.5 ** (0.4, 0.6)	0.4 ** (0.3, 0.5)	<0.001

Results from logistic regression analysis given as odds ratio (95% CI). Adjusted for age groups (40–49 years (reference)/50–59 years/60–69 years/70–79 years/80–96 years), body mass index groups (normal (reference)/overweight/obesity), physical activity level (sedentary (reference)/light/moderate/vigorous) and smoking status (never smoker (reference)/current smoker/previous smoker). * *p*-value for logistic regression analysis <0.05. ** *p*-value for logistic regression analysis <0.001. CI, confidence interval. E%, proportion of total energy intake. **^a^** 1: Primary education (up to 10 years), 2: upper secondary education (minimum 3 years), 3: tertiary education, short: college/university less than 4 years, 4: tertiary education, long: college/university 4 years or more.

## Data Availability

The data supporting the results in this paper is available through application to the Tromsø Study at UiT The Arctic University of Norway (https://uit.no/research/tromsoundersokelsen). Data are not publicly available.

## Supplementary Materials

The following are available online at https://www.mdpi.com/2072-6643/13/2/405/s1, Table S1: Characteristics of participants included and excluded in the final study sample. The Tromsø Study 2015–2016.

## References

[1] Wilkinson R.G., Marmot M.G. (2006). Social Determinants of Health.

[2] Strand B., Tverdal A. (2006). Trends in educational inequalities in cardiovascular risk factors: A longitudinal study among 48,000 middle-aged Norwegian men and women. Eur. J. Epidemiol..

[3] Eggen A.E., Mathiesen E.B., Wilsgaard T., Jacobsen B.K., Njølstad I. (2014). Trends in cardiovascular risk factors across levels of education in a general population: Is the educational gap increasing? The Tromsø study 1994–2008. J. Epidemiol. Community Health.

[4] Marmot M., Friel S., Bell R., Houweling T.A., Taylor S. (2008). Closing the gap in a generation: Health equity through action on the social determinants of health. Lancet.

[5] Gallo V., Mackenbach J.P., Ezzati M., Menvielle G., Kunst A.E., Rohrmann S., Kaaks R., Teucher B., Boeing H., Bergmann M.M. (2012). Social Inequalities and Mortality in Europe–Results from a Large Multi-National Cohort (Social Inequalities and Mortality in Europe). PLoS ONE.

[6] World Health Organization-Regional office for Europe (2019). Healthy, Prosperous Lives for All: The European Health Equity Status Report.

[7] Sustainable Development Goal 10. https://www.un.org/sustainabledevelopment/inequality/.

[8] World Health Organization (2013). Global Action Plan for the Prevention and Control of Noncommunicable Diseases 2013–2020.

[9] Afshin A., Sur P.J., Fay K.A., Cornaby L., Ferrara G., Salama J.S., Mullany E.C., Abate K.H., Abbafati C., Abebe Z. (2019). Health effects of dietary risks in 195 countries, 1990–2017: A systematic analysis for the Global Burden of Disease Study 2017. Lancet.

[10] Totland T.H., Melnæs B.K., Lundberg-Hallen N., Helland-Kigen K.M., Lund-Blix N.A., Myhre J.B., Johansen A.M.W., Løken E.B., Andersen L.F. (2012). NORKOST 3-En Landsomfattende Kostholdsundersøkelsen Blant Menn og Kvinner i Norge i Alderen 18-70 år, 2010–2011.

[11] Laursen U.B., Johansen M.B., Joensen A.M., Lau C.J., Overvad K., Larsen M.L. (2019). Educational level and living arrangements are associated with dietary intake of red meat and fruit/vegetables: A Danish cross-sectional study. Scand. J. Public Health.

[12] Ovaskainen M.-L., Paturi M., Tapanainen H., Harald K. (2010). Educational differences in the diet of Finnish adults and the associations between education and the determinants and facilitators of dietary fat quality. Public Health Nutr..

[13] Jacobsen B.K., Nilsen H. (2000). High education is associated with low fat and high fibre, beta-carotene and vitamin C computation of nutrient intake based on a short food frequency questionnaire in 17,265 men and women in the Tromsø study. Nor. J. Epidemiol..

[14] Nordic Council of Ministers (2014). Nordic Nutrition Recommendations 2012: Integrating Nutrition and Physical Activity.

[15] Amcoff E., Edberg A., Barbieri H.E., Lindroos A.K., Nälsén C., Pearson M., Lemming E.W. (2012). Riksmaten–vuxna 2010–11. Livsmedels- och näringsintag bland vuxna i Sverige.

[16] Pedersen A.N., Christensen T., Matthiessen J., Knudsen V.K., Sørensen M.R., Biltoft-Jensen A., Fagt S. (2015). Danskernes kostvaner 2011–2013-Hovedresultater.

[17] Helldán A., Raulio S., Kosola M., Tapanainen H., Ovaskainen M.-L., Virtanen S. (2013). The National FINDIET 2012 Survey.

[18] Tromsøundersøkelsen-Tromsø 7 The Tromsø Study-Tromsø 7. https://uit.no/research/tromsoundersokelsen/project?pid=706786.

[19] Carlsen M.H., Lillegaard I.T.L., Karlsen A., Blomhoff R., Drevon C.A., Andersen L.F. (2010). Evaluation of energy and dietary intake estimates from a food frequency questionnaire using independent energy expenditure measurement and weighed food records. Nutr. J..

[20] Carlsen M.H., Karlsen A., Lillegaard I.T., Gran J.M., Drevon C.A., Blomhoff R., Andersen L.F. (2011). Relative validity of fruit and vegetable intake estimated from an FFQ, using carotenoid and flavonoid biomarkers and the method of triads. Br. J. Nutr..

[21] Lundblad M.W., Andersen L.F., Jacobsen B.K., Carlsen M.H., Hjartåker A., Grimsgaard S., Hopstock L.A. (2019). Energy and nutrient intakes in relation to National Nutrition Recommendations in a Norwegian population-based sample: The Tromsø Study 2015–16. Food Nutr. Res..

[22] Norwegian Food Composition Database 2018. https://matvaretabellen.no/?language=en.

[23] Grimby G., Börjesson M., Jonsdottir I.H., Schnohr P., Thelle D.S., Saltin B. (2015). The “Saltin–Grimby Physical Activity Level Scale” and its application to health research. Scand. J. Med. Sci. Sports.

[24] Paturi M., Tapanainen H., Reinivuo H., Pietinen P. (2008). The National FINDIET 2007 Survey.

[25] Blakely T., Cleghorn C., Mizdrak A., Waterlander W., Nghiem N., Swinburn B., Wilson N., Mhurchu C.N. (2020). The effect of food taxes and subsidies on population health and health costs: A modelling study. Lancet Public Health.

[26] Educational Attainment of the Population. https://www.ssb.no/en/utdanning/statistikker/utniv/aar.

[27] Population, by Sex and Age Groups 2019. https://www.ssb.no/en/befolkning/statistikker/folkemengde/aar-per-1-januar.

[28] Statbank-Tobacco, Alcohol and Other Drugs 05307: Percentage Daily Smokers and Occasional Smokers, by Sex and Age (Per cent) 1973–2019. https://www.ssb.no/en/statbank/table/05307.

[29] Jacobsen B.K., Thelle D.S. (1988). The Tromsø Heart Study: Responders and Non-responders to a Health Questionnaire, Do They Differ?. Scand. J. Soc. Med..

[30] Knudsen A.K., Hotopf M., Skogen J.C., Øverland S., Mykletun A. (2010). The Health Status of Nonparticipants in a Population-based Health Study: The Hordaland Health Study. Am. J. Epidemiol..

[31] Langhammer A., Krokstad S., Romundstad P., Heggland J., Holmen J. (2012). The HUNT study: Participation is associated with survival and depends on socioeconomic status, diseases and symptoms. BMC Med. Res. Methodol..

[32] Ferruzzi M., Delahanty L., Coulston A.M., Boushey C.J., Johnson R.K., Kerr D.A., Schap T.E. (2017). Analysis, Presentation, and Interpretation of Dietary Data. Nutrition in the Prevention and Treatment of Disease.

[33] Elstad J.I. (2008). Utdanning og Helseulikheter-Problemstillinger og Forskningsfunn. Education and Inequalities in Health.

[34] Si Hassen W., Castetbon K., Cardon P., Enaux C., Nicolaou M., Lien N., Terragni L., Holdsworth M., Stronks K., Hercberg S. (2016). Socioeconomic indicators are independently associated with nutrient intake in French adults. Nutrients.

[35] Carlsen M.H., Blomhoff R., Andersen L.F. (2011). Intakes of culinary herbs and spices from a food frequency questionnaire evaluated against 28-days estimated records. Nutr. J..

[36] Johansson L., Solvoll K. (1999). NORKOST 1997. Statens råd for Ernæring og Fysisk Aktivitet.

[37] Rylander C., Sandanger T.M., Engeset D., Lund E. (2014). Consumption of lean fish reduces the risk of type 2 diabetes mellitus: A prospective population based cohort study of Norwegian women. PLoS ONE.

[38] Bakken T., Braaten T., Olsen A., Kyrø C., Lund E., Skeie G. (2016). Consumption of Whole-Grain Bread and Risk of Colorectal Cancer among Norwegian Women (the NOWAC Study). Nutrients.

[39] Social Desirability in the Encyclopedia of Survey Research Methods. https://methods.sagepub.com/reference/encyclopedia-of-survey-research-methods.

