# Supplementary material for: The Educational Gradient in Intake of Energy and Macronutrients in the General Adult and Elderly Population: The Tromsø Study 2015–2016
Source: Nutrients. 2021 Jan 28;13(2):405. doi: 10.3390/nu13020405 (PMC7911135; doi:10.3390/nu13020405)
Supplement: Supplementary file 1 [file nutrients-13-00405-s001.pdf]

Table S1: Characteristics of participants included and excluded in the final study sample. The Tromsø Study 2015-2016.

		**Attended Tromsø 7 (N=21,083)**				
		**Included in final sample (n=11,302)**		**Excluded (n=9781)**		
		n	Mean (SD) or proportion	n	Mean (SD) or proportion	*p*
Sex	Women	6043	53.5	5031	51.4	<0.05
	Men	5259	46.5	4750	48.6	
Age (years)		11302	57.4 (10.8)	9781	57.3 (12.1)	0.5
Age-groups	40-49 years	3256	28.8	3176	32.5	<0.001
	50-59 years	3254	28.8	2781	28.4	
	60-69 years	3131	27.7	2048	20.9	
	70-79 years	1405	12.4	1271	13.0	
	80+ years	256	2.3	505	5.2	
BMI (kg/m^2)		11274	27.2 (4.5)	9746	27.5 (4.6)	<0.001
BMI-group[a]	Normal	3749	33.3	3002	30.8	<0.001
	Overweight	4950	43.9	4252	43.6	
	Obesity	2575	22.8	2492	25.6	
Education level[b]	Primary	2327	20.6	2469	26.3	<0.001
	Upper secondary	3064	27.1	2692	28.6	
	Tertiary, short (<4 years)	2321	20.5	1687	17.9	
	Tertiary, long (≥ 4 years)	3590	31.8	2555	27.2	
Physical activity level[c]	Sedentary	1439	13.0	1533	16.5	<0.001
	Light	6512	58.8	5303	57.1	
	Moderate	2791	25.2	2160	23.2	
	Vigorous	334	3.0	298	3.2	
Smoking status	Never smokers	4788	42.6	3945	40.9	<0.001
	Previous smokers	5043	44.8	4207	43.6	
	Current smokers	1415	12.6	1489	15.4	

Differences between the included and excluded participants were tested by Student´s t-test (for continuous variables) and Pearson´s chi-square test (for categorical variables)

SD, standard deviation. BMI, body mass index.

[a] Normal (BMI < 25.0 kg/m^2), overweight (BMI 25.0-29.9 kg/m^2), obesity (BMI ≥ 30 kg/m^2)

[b] Primary education (up to 10 years), Upper secondary education (minimum 3 years), Tertiary education, short: college/university less than 4 years, Tertiary education, long: college/university 4 years or more.

[c] Exercise and physical activity in leisure time over the last year. Sedentary: reading, watching TV/screen or other sedentary activity, Light: walking, cycling or other forms of exercise at least 4 hours a week, Moderate: participation in recreational sports, heavy gardening, snow shoveling etc. at least 4 hours a week, Vigorous: participation in hard training or sports competitions, regularly several times a week
